# Vascular anomalies: special considerations in children

**DOI:** 10.1186/s42155-020-00153-y

**Published:** 2020-11-22

**Authors:** Craig R. Gibson, Alex M. Barnacle

**Affiliations:** 1 Department of Medical Imaging, Perth Children’s Hospital, Perth, Western Australia Australia; 2 Department of Radiology, Great Ormond Street Hospital for Children, London, WC1N 3JH UK

**Keywords:** Vascular malformation, Haemangioma, Paediatric, Fibroadipose vascular anomaly, Venous malformation, Lymphatic malformation, Sclerotherapy, Embolisation

## Abstract

The diagnosis and treatment of vascular anomalies are a large part of the caseload for paediatric interventional radiologists. Although many of the principles of sclerotherapy and embolisation are the same in adult and paediatric practice, there are some key differences in the approach for children, including some longer term thinking about managing these chronic diseases and their impact on a growing child. Vascular tumours are not often seen in adult IR practice and the rarest can be life threatening; knowledge of the commonest types and the role IR can play in their management can be instrumental in ensuring that children get appropriate treatment in a timely manner. Vascular anomalies also encompass some conditions associated with complex overgrowth, a subject that often causes confusion and uncertainty for interventional radiologists. This paper presents a simplified and practical approach to this spectrum of disease.

## Background

The term vascular anomalies incorporates a diverse range of pathologies which are best understood when classified as either vascular tumours or vascular malformations. The 2018 guidelines published by The International Society for the Study of Vascular Anomalies (ISSVA 2018) breaks this down into significantly more detailed categories although several pathologies have considerable overlapping clinical and imaging characteristics (ISSVA 2018; Dasgupta and Fishman 2014). Although vascular anomalies are relatively common, their correct diagnosis and management is notoriously poorly understood, which is a source of frustration for patients and risk for their clinicians (Patel and Barnacle 2017). This is particularly the case for children, where parental anxieties are often heightened.

Vascular anomalies in children warrant special consideration for a multitude of reasons. Families will often have seen many other teams and been given a variety of diagnostic labels and misleading advice. Parents or care givers will have questions over the correct diagnosis and what it means for their child over the course of their childhood. They often have pre-formed views on whether their child should be treated or not and many will initially push for a surgical ‘cure’. Their opinions are often strongly influenced by concerns over the physical appearance of their child, worries over genetic implications and guilt over a delayed diagnosis. Explaining a complex condition and suggesting a treatment which rarely has defined outcome measures is a challenge. How do we determine the most appropriate time to intervene, balancing the patient’s age against potential developmental impairment? How do we make sure that we are genuinely treating the patient and not the parents? And how do we ensure the child is empowered by and invested in the decisions we make?

Of course, the answers to these questions come in every shade of grey, which makes the management of vascular anomalies in children both fascinating and challenging.

### Vascular tumours

Involvement in the management of benign vascular tumours may be a relatively rare occurrence for an interventional radiologist (IR). However, a working knowledge of this disparate group of pathologies is crucial in the following settings:

- Identifying benign vascular tumours when they present and understanding their natural history gives IRs the ability to reassure families and clinicians and to refer the child on for appropriate medical intervention when needed
- Recognising a child presenting with a rare and clinically aggressive high flow vascular tumour allows IRs to push for urgent and often life-saving endovascular treatment
- Appreciating atypical features suggestive of an alternative diagnosis such as malignancy means that IRs are often the first to raise concerns and recommend biopsy.

ISSVA (2018) divides vascular tumours into benign, locally aggressive/ borderline and aggressive tumours. A complete overview of these pathologies is beyond the remit of this paper but a thorough understanding of haemangiomas is essential and one other subtype of vascular tumour merits brief mention.

Haemangiomas are broadly classified as infantile or congenital; the infantile subtype is much more common. As their names suggest, infantile haemangiomas (IHs) develop during infancy, (a proportion may be present as a subtle punctate lesion at birth but the majority of their growth is postnatal); while congenital haemangiomas (CHs) are fully developed at birth (Krol and MacArthur 2005).

**Fig. 1:**
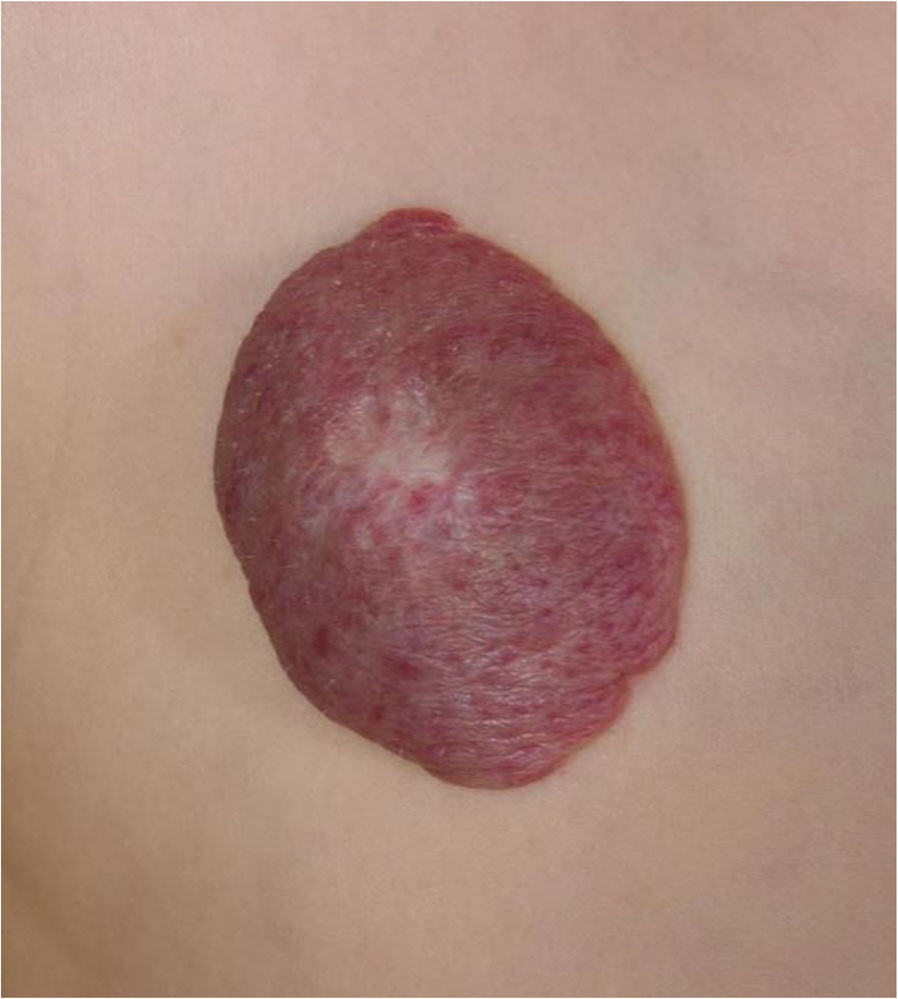
Typical appearance of a superficial infantile haemangioma

IHs have a highly characteristic growth pattern, consisting of a rapid proliferative phase usually from 4 to 6 weeks of age and lasting 6–12 months, followed by a plateau in growth and then a much slower involutional phase, resulting in spontaneous resolution of > 90% of tumours by the age of 10 years (Fig. 1). Recognition of this pattern is key although if there is any clinical doubt, biopsy of an IH will specifically immunostain for the glucose transporter protein GLUT-1 (Smith et al. 2017). In children where the tumour is in a particularly disfiguring or disabling position, such as those involving the periorbital soft tissues or airway, oral beta-blockers are highly effective in curbing growth if given during the proliferative phase (Hogeling et al. 2011; Hoeger et al. 2015). Note that IHs are the only vascular tumour that responds to propranolol.

**Fig. 2:**
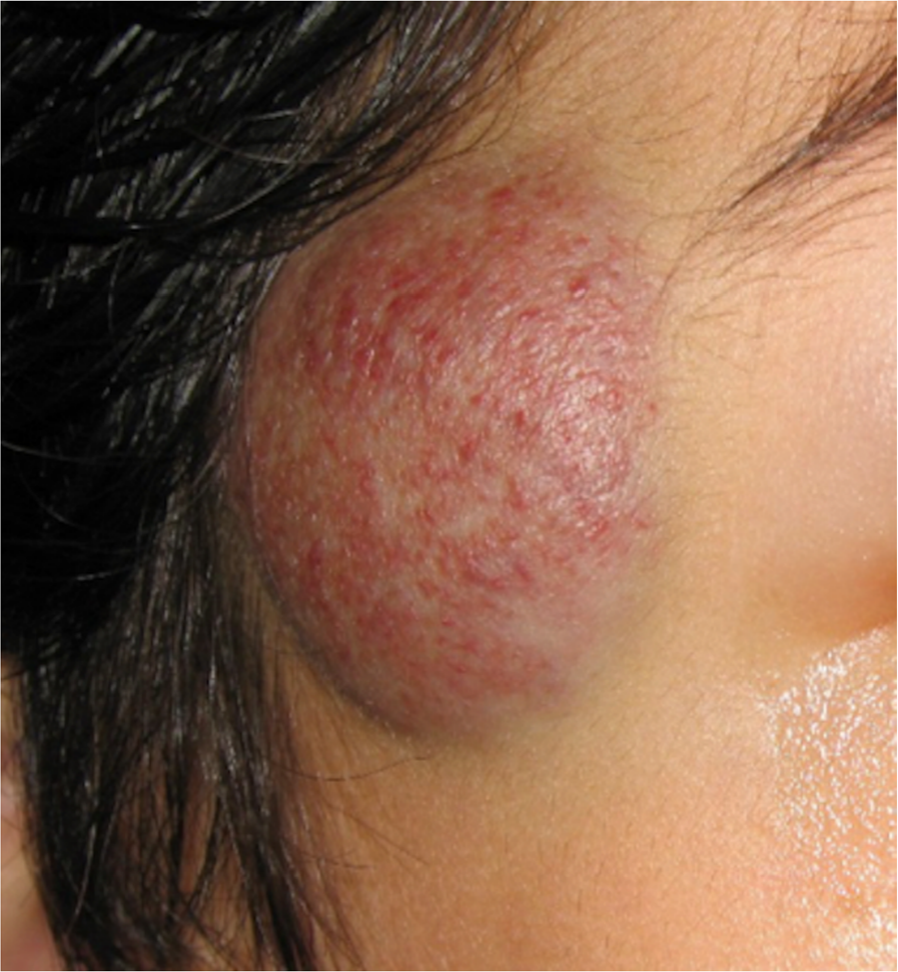
Classic appearance of a non-involuting congenital haemangioma (NICH). They have a more purple colour than the commoner infantile haemangiomas

**Fig. 3:**
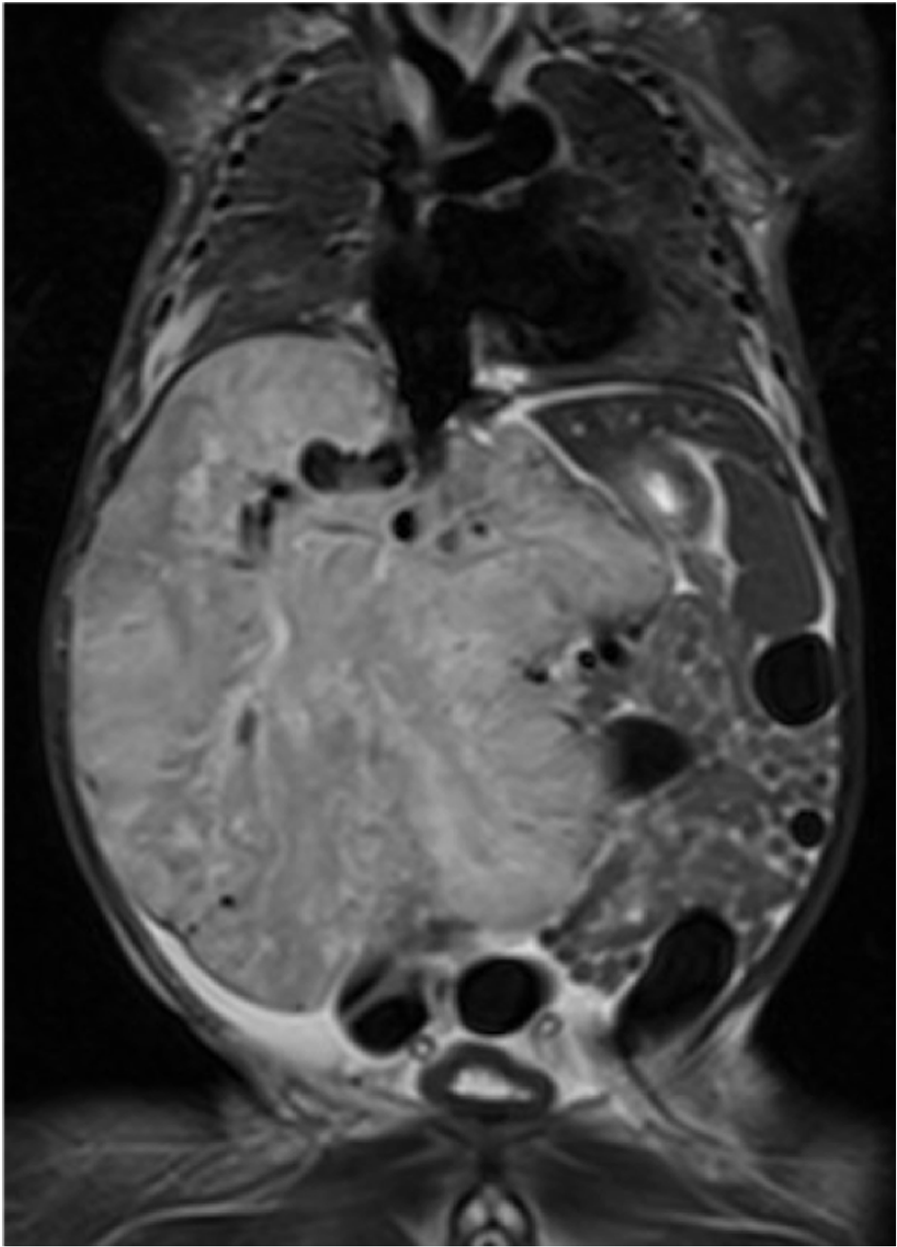
Coronal T2 weighted MR image, showing a large rapidly-involuting congenital haemangioma (RICH) occupying almost all of the liver in a 3-day old male. The lesion caused splinting of the diaphragm, high output cardiac failure and abdominal compartment syndrome

CHs are far less common. They look different to IHs, with a more purple colour and often an overlying telangiectasia and a pale halo (Fig. 2). Within this group, there are distinct clinical variants: rapidly involuting (RICH), partially involuting (PICH) and non-involuting (NICH) subtypes, named according to their growth patterns (Krol and MacArthur 2005). RICHs are, by nature, transient lesions which usually resolve entirely by 6–12 months of life without any intervention. However, they can be life threatening in the first few weeks of life due to their propensity to present at birth as huge tumours with associated arteriovenous shunting, causing overwhelming heart failure in the new-born (Fig. 3). Most infants with medically significant lesions can be medically supported in the first few weeks of life and intervention is rarely necessary. However, very large lesions causing severe cardiac failure require urgent embolisation to reduce the shunting until the lesion’s natural involution occurs. Such intervention is rare but often lifesaving. RICHs can be associated with thrombocytopenia in the acute phase, due to platelet damage or consumption in their large vascular bed. This is often mistaken for Kasabach-Merritt phenomenon, a process only seen in association with another vascular tumour type (see below). The thrombocytopaenia will not be corrected by platelet transfusion; indeed, transfusions will cause further consumption and swelling of the tumour. The platelet count recovers as the tumour shrinks.

NICHs tend to be much smaller and asymptomatic. Typically, they don’t regress but may become less obvious over time.

PICHs represent a small subset of CHs which demonstrate intermediate behaviours somewhere between the two extremes represented by RICHs and NICHs. (Nasseri et al. 2014).

Disseminated haemangiomatosis is a term used to describe multiple cutaneous and visceral infantile haemangiomas. Numerous liver lesions, which may lead to compartment syndrome, impaired liver function and cardiac failure, require prompt and decisive treatment with propranolol (Glick et al. 2012).

Although haemangiomas are much more frequently isolated mass lesions, in rare instances they can be part of a more extensive cluster of abnormalities (Smith et al. 2017). PHACE syndrome is the most well-known of these. It is a neurocutaneous syndrome which consists of a rather characteristic flat facial or scalp infantile haemangioma (H) plus at least one of the following structural abnormalities: (P) posterior fossa malformations, (A) arterial anomaly, (C) cardiac defects or coarctation and (E) eye anomalies (Garzon et al. 2016).

**Fig. 4:**
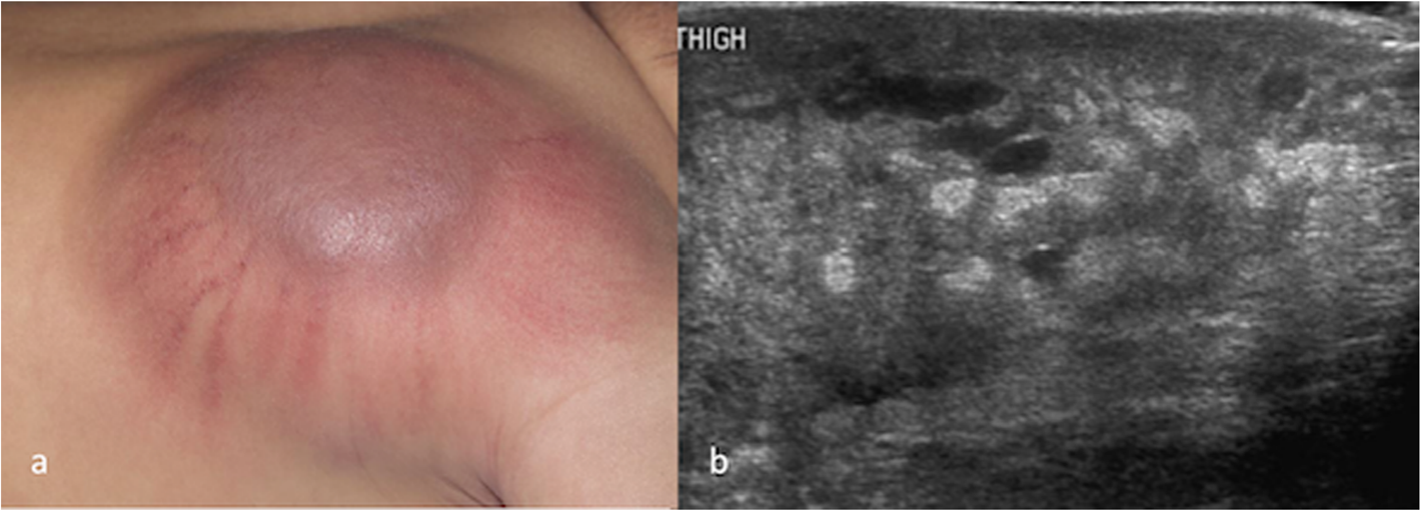
**a** a kaposiform haemangioendothelioma (KHE) in a 1 month old female. Clinically, the lesion appears more aggressive in nature than the common infantile haemangiomas; **b** an ultrasound image of a KHE in another patient, showing a far more heterogenous parenchymal echotexture than that seen in haemangiomas

It is worth mentioning two rarer vascular tumour types, tufted angiomas and kaposiform haemangioendotheliomas (KHE), because of their association with the potentially life-threatening Kasabach-Merritt Phenomenon (KMP) (Mahajan et al. 2017). KMP describes a profound thrombocytopaenia with low fibrinogen and raised D-dimers. Like IHs, both of these tumours, which histologically represent ends of the same spectrum, tend to appear in early childhood, are usually in a cutaneous/subcutaneous location. They look dissimilar to haemangiomas both clinically and on imaging and are GLUT-1 negative on immunostaining (Fig. 4). Biopsy is the gold standard for diagnosis due to their characteristic histopathological features. They can be painful, unlike other vascular tumours, and when associated with KMP they demonstrate the sequelae of the consumptive coagulopathy and severe thrombocytopaenia such as widespread petechiae and bleeding. Without treatment of the lesion, the coagulopathy can result in significant morbidity and mortality. Until recently, vincristine was the treatment of choice for KHE and tufted angiomas but sirolimus is now more commonly used (Tasani et al. 2017).

### Vascular malformations

Vascular malformations are present from birth, though often not clinically obvious until later. Low flow malformations present more frequently in children than high flow lesions, the latter often not becoming symptomatic until teenage years. Although many of the principles of sclerotherapy and embolisation are the same in adult and paediatric practice, there are some key differences in the approach for children, including some longer term thinking about managing these chronic diseases and their impact on a growing child. Most interventionalists would argue that the primary indication for active intervention is to improve function for a patient limited by a vascular malformation. In paediatrics, the decision-making can be a little more complicated. A child or family’s desire for a “better” cosmetic appearance should be approached with a degree of caution. Undoubtedly this can be a valid indication when paired with altered self-confidence or self-image and bullying, especially when a malformation is in a very visible location. Smolak suggests that children start to become body conscious around the age of 6 years, and between 40 and 50% of children of ages of 6–12 express some dissatisfaction with a component of their appearance (Smolak 2011). By this age it is therefore reasonable to infer that children are able to indicate their own preference regarding their desire for treatment. We must ensure that a) we are not purely treating the parents and their wish for a “perfect child” and b) that we manage a child’s expectations in terms of the anticipated outcome.

Children with extensive vascular malformations will require repeated interventions; explaining this and presenting it in a practical and positive way is an important part of helping children and families to come to terms with the diagnosis of a chronic illness and facilitating compliance with future procedures. The best outcomes and patient experiences are achieved by utilising an expert and proactive multidisciplinary team. Dermatologists are key not only for their expertise in managing skin lesions but they are often the clinicians with the greatest understanding of the medical and genetic implications of these diseases; they and paediatricians or oncologists usually have a central role in the medical management of vascular anomalies. Regional clinics should have access to specialist genetics services, as these teams are key to looking for underlying mutations that may lead to a unifying diagnosis for children with complex problems and in highlighting genetic anomalies that may prove to be therapeutic targets for novel therapies. Active engagement from orthopaedic and plastic surgeons is vital to manage aspects of overgrowth and other surgical specialties are required for site-specific disease, such as ear, nose and throat surgeons, urologists, gastrointestinal surgeons and oculoplastic surgeons. Children with painful malformations, such as venous malformations causing destructive arthritis, should be offered access to a chronic pain management service. A host of other specialists are also needed to manage functional issues. These include physiotherapists, speech and occupational therapists and, importantly, psychologists to help patients deal with often profound concerns surrounding differences in appearance. IRs can be key coordinators of this team, with our diagnostic expertise in imaging vascular anomalies and our ability to contribute to the treatment of so many of these conditions. Developing our own clinics and being a regular presence in our colleagues’ clinics increases our influence. These rare and complex conditions challenge us to become more holistic doctors, giving careful consideration to each one of a patient’s complex needs.

### Venous malformations

Venous malformations (VMs) are the most common of the slow-flow vascular malformations. They result from errors during venous embryogenesis (Dompmartin et al. 2010). They have a range of morphologies, from well-defined, compressible spongiform lesions (a morphology classified as Puig type I) to a complex tangle of dysplastic veins (Puig type IV) (Puig et al. 2003). In many instances VMs are asymptomatic but, depending on their location, can present with pain, thrombosis, functional impairment and cosmetic issues. Although present from birth, they may only become apparent later in life if not superficial in location (Hassanein et al. 2012). VMs commonly become more symptomatic for children during puberty, enlarging during growth spurts.

Larger VMs can be associated with a coagulopathy, a complication that is under-recognised in this disease (Dompmartin et al. 2008; Zhuo et al. 2017). Blood stagnation within abnormal venous channels repeatedly activates a low-grade coagulation cascade, leading to the continuous depletion of clotting factors, including fibrinogen. In the rare patients with an extensive VM, this can result in a severe systemic coagulopathy, with fibrinogen levels < 1.0, low factor XIII and extremely high D-dimers. An extended clotting profile should be checked in patients with extensive VMs and those patients affected should know to warn surgeons of their coagulopathy prior to any surgery. Experienced haematology management is essential. Severe coagulopathies can, counter-intuitively, be improved to some degree by daily low-molecular weight heparin administration, which reduces the frequency of pointless clot formation within the VM (Zhuo et al. 2017). Aspirin has no effect.

As with VMs in adults, treatments strategies include compression garments, sclerotherapy and surgery. Depending on the site and the symptomatology of the VM conservative management may suffice. That may entail simple reassurance that a small, innocuous, pain free VM will not evolve into a more aggressive lesion. If venous congestion, pain or mass effect is an issue, then a well-tailored compression garment can be a simple, non-invasive means of symptom control (Langbroek et al. 2016). These garments work by flattening out the venous lakes or patulous vessels to prevent blood stasis, reducing swelling and painful thrombosis. They are, however, only effective while in place. They are an excellent intervention in dependent lesions or for patients whose symptoms are only exacerbated by activity.

**Fig. 5:**
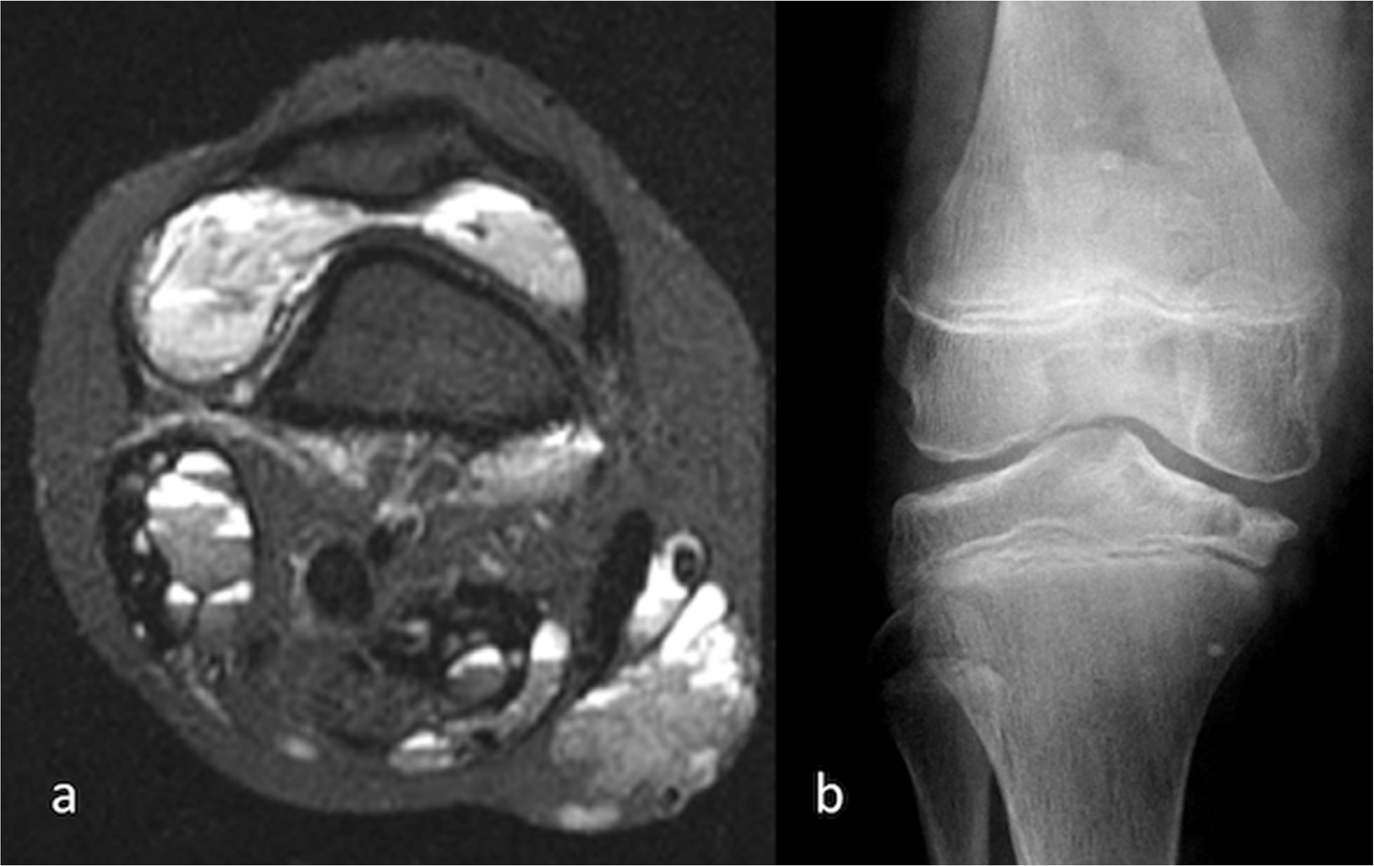
Venous malformation (VM) of the knee in a 10 year old female. **a** Axial T2 weighted MR image shows VM within the soft tissues around the knee and within the knee joint itself; **b** AP radiograph confirms severe articular damage to the knee joint. Soft tissue thickening and phleboliths are also noted

Sclerotherapy is generally accepted as the first line active intervention for VMs. It can shrink smaller lesions very effectively and is usually an excellent means of symptom control for more extensive lesions. It aims to scar and close the venous channels, thus alleviating venous congestion and thrombosis and reducing mass effect (Clemens et al. 2017). In children, there are additional factors to consider. VMs with an intra-articular component often cause recurrent haemarthroses and, in a similar manner to haemophilia, can result in a destructive arthropathy (Hu et al. 2018) (Fig. 5). These are most commonly seen around the knee. Avoidance of this devastating complication in young children is a compelling indication for early intervention, even on asymptomatic joints.

The choice of sclerosing agents in children is the same as that in adults but extra attention must be paid to dose limits and potential side effects. A full description of the sclerosants available and their various properties is beyond the scope of this paper but there are some factors that merit discussion.

Ethanol was historically the commonest sclerosing agent used but its high complication rate and dose limitations (see below) have made it less popular in recent years. It is perhaps the most effective agent, causing direct vessel wall necrosis and disruption of erythrocytes, with subsequent thrombosis and fibrosis of the intima. The dose administered is severely limited by local and systemic side effects; a dose of 1.0 mL/kg may cause respiratory depression, cardiac arrhythmia, rhabdomyolysis and even sudden death (Qiu et al. 2013; Yakes 2004; Alomari and Dubois 2011). Local side effects include skin necrosis and nerve damage.

Sodium tetradecyl sulfate (STS) is now the most widely used agent. It is highly effective due to its detergent properties, which interfere with cell surface lipids and produce maximum endothelial damage with minimal thrombus formation leading to fibrosis of the lesion and eventually to shrinkage of the lesion (Clemens et al. 2017). In children, it is reasonable to work on a maximum STS 3% dose per procedure of 0.5 ml/kg, up to a maximum of around 20mls (Burrows and Mason 2004). It is well recognised by centres that perform sclerotherapy regularly, and in the scientific literature, that some patients develop discoloured urine within a few hours of STS sclerotherapy (Stepien et al. 2011; Barranco-Pons et al. 2012). This phenomenon is described in many published sclerotherapy papers and is assumed to be due to haemoglobinuria. Urine discolouration and oliguria are observed more commonly after higher doses of STS have been administered (Barranco-Pons et al. 2012). Renal injury is usually transient but can lead to end-stage renal failure and the need for dialysis. To avoid risk of renal injury, peri-operative measures such as intravenous hydration and bladder catheterisation have been suggested by some authors but have not been universally agreed, and practice varies widely between centres (Barranco-Pons et al. 2012). The simplest way to minimise the risk of renal injury is by keeping within safe dose limits.

The dose of STS should also be tailored to the location and morphology of the lesion. For instance, a large, spongiform, intramuscular quadriceps lesion may take a relatively large dose but a small digital lesion will tolerate only a fraction of that dose (in a child of any size) before risking local complications. STS can cause significant swelling, which should be taken into account in tight spaces such as the carpel tunnel or in the face. It is associated with significant side effects and should be used judiciously. These include skin ulceration, nerve injury and DVT (Burrows and Mason 2004; Stuart et al. 2015).

STS is routinely administered as a foam. There is wide variation in the make-up of STS foam. The sclerosant is agitated with air, contrast medium, ethiodized oil or albumin solution to create a thickened solution that fills the malformation mores slowly and is more likely to gain 360^0^ wall contact for effective endothelial damage. It has the added benefit in children of giving a higher volume of agent for the same STS dose (Rabe and Pannier 2013).

Other sclerosing agents are available. Polidocanol is a synthetic long-chain fatty alcohol which is also injected as a foam. It is essentially a weaker detergent-type of sclerosing solution than STS and its treatment and side effect profile are both considered to be slightly diminished (McAree et al. 2012).

**Fig. 6:**
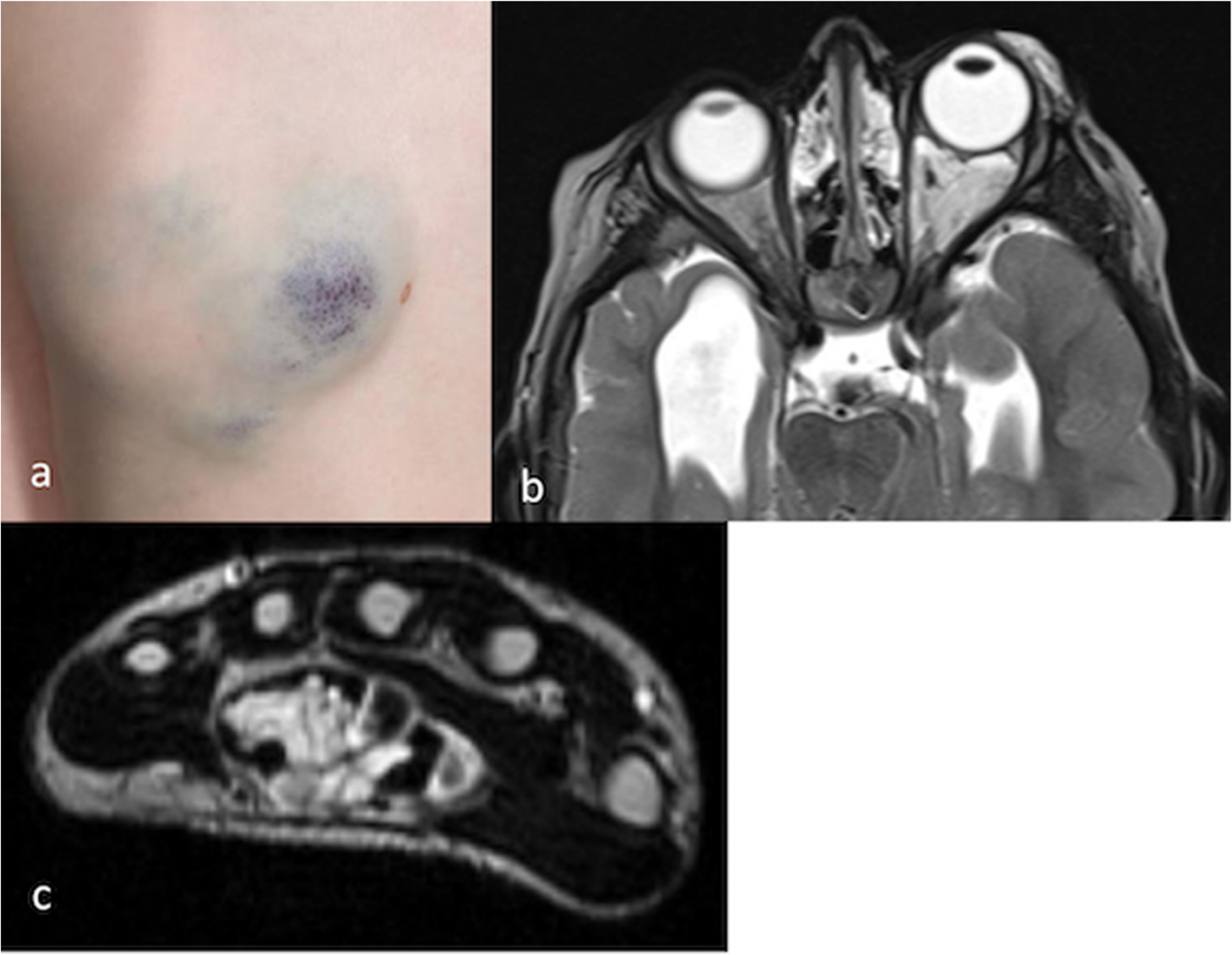
Venous malformations (VMs) that may be better suited to bleomycin sclerotherapy than with other agents **a** VM with a superficial component involving the dermis, which is likely to blister with other agents; **b** axial T2 weighted MR image of a VM of the left posterior orbit and eyelid in a 3 year old female. Intracranial abnormalities are also noted; **c** axial T2 weighted MR image of the wrist in a 14 year old female. The VM lies within the carpal tunnel itself

Bleomycin has a different mechanism of action. It appears to break down the solid stroma of a VM via its cytotoxic action, though its exact mechanism of sclerosis is still unclear (Chaudry et al. 2014). It typically causes less swelling and is not known to be neurotoxic. It very rarely causes skin breakdown, so it is an attractive agent to use near nerves and in tight spaces and superficial lesions (Fig. 6). It seems most effective in relatively spongy VMS with a large stromal component suggesting that injections should perhaps specifically be aimed at the solid components. Mixing bleomycin with agents such as 20% albumin, ethiodized oil or gelatin sponge slurry may reduce the rate of wash-out of the agent from the VM, which may increase its efficacy.

Puig et al. established a classification for VMs based on appearance and venous drainage with subsequent implications regarding the likelihood of a successful response to sclerotherapy (Puig et al. 2003). Simply put, VMs demonstrating minimal outflow (Puig type I and II) are associated with better outcomes primarily because the sclerosant stays within the target lesion for longer. Relatively rapid venous drainage from Puig type III and IV VMs represents a challenge for IRs as systemic escape of the sclerosant both impedes the success of sclerotherapy and exposes the patient to the potential for systemic effects of the sclerosant. In these cases, venous outflow obstruction during the procedure should be considered. This may simply be manual by extrinsically placing pressure over the outflow tract or it may be more sophisticated by using glue or coils to internally obstruct the outflow vessels (Legiehn 2019; Chewning et al. 2018) (Fig. 6).

In patients with relatively solid VMs resistant to sclerotherapy and not amenable to surgery, there is emerging evidence to suggest that percutaneous cryoablation may represent a viable and safe alternative for debulking large lesions (Cornelis et al. 2017).

#### Blue rubber bleb naevus syndrome (BRBNS)

**Fig. 7:**
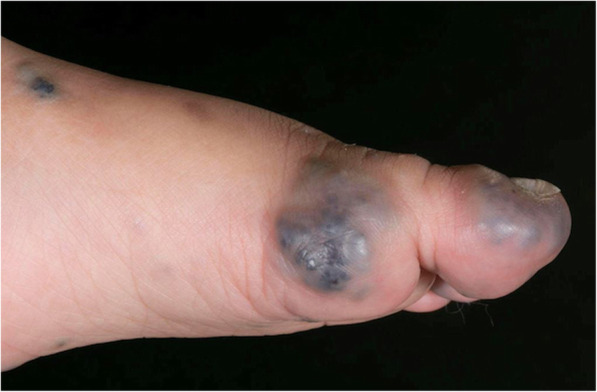
Multiple cutaneous lesions with the classic features of blue rubber bleb naevi in a 2 year old male

Blue rubber bleb naevus syndrome (BRBNS) is a genetic disorder linked to *TEK* or TIE2 mutations. It is characterised by multiple small cutaneous and visceral venous malformations, often with involvement of the gastrointestinal tract, which is considered pathognomonic (Soblet et al. 2017) (Fig. 7). Extensive BRBNS can be challenging to manage, particularly bleeding from the gut which can lead to iron deficiency anaemia and transfusion-dependence. Anecdotal evidence suggests the superficial malformations are less responsive to sclerotherapy than typical VMs. Sirolimus appears to have a role in managing symptomatic gut lesions (Wang et al. 2020; Wong et al. 2019).

### Lymphatic malformations

**Fig. 8:**
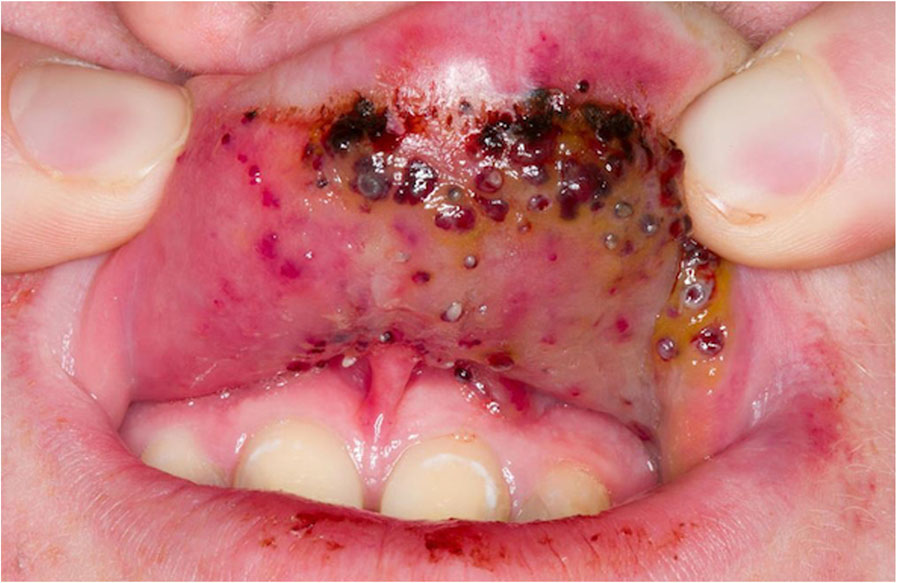
Characteristic appearance of infected or inflamed lymphatic vesicles in a teenage child with a lymphatic malformation of the buccal mucosa

**Fig. 9:**
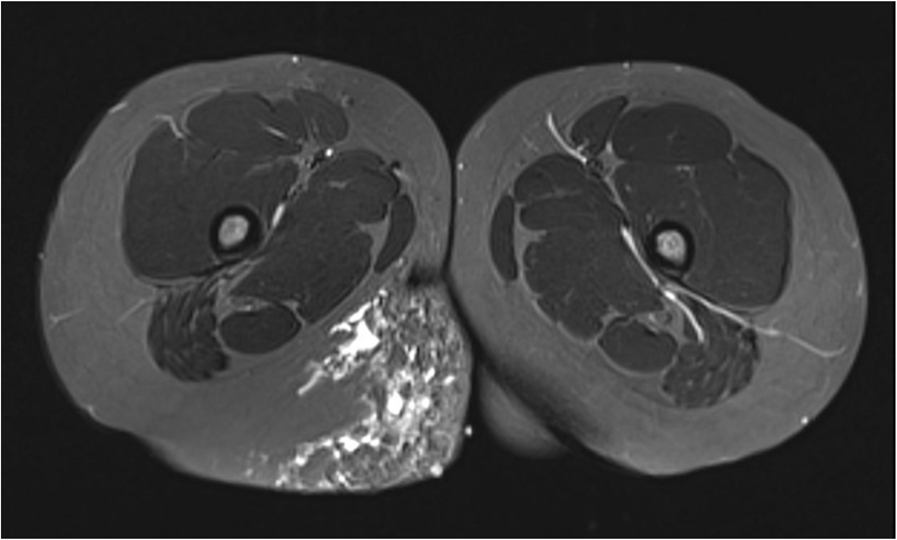
Axial T2 weighted MR image of a 9 year old male with a lymphatic malformation of the right buttock. The subcutaneous lymphatic cysts, which are of high T2 signal, are of varying sizes and there is additional fat present, contributing to the bulk of the lesion

Patients with lymphatic malformations (LMs) are seen far more commonly in a paediatric IR clinic than in adult practice probably because these lesions present in early childhood, can cause parental alarm and are usually treated before patients are transitioned to adult care. LMs can be considered to be a collection of thin-walled cysts containing lymphatic fluid. Some key behavioural features which may aid in diagnosis are their propensity to swell during concurrent viral illnesses and recurrent swelling associated with bruising due to repeated intralesional haemorrhage. Some patients are prone to localised cyst infection, particularly if the lesion affects mucosa. When it affects the skin or mucosa, it appears as fine surface nodules, often with punctate red or black markings (Fig. 8); skin lesions often leak lymphatic fluid. Microcystic disease can be associated with localised fat hypertrophy (Fig. 9). This is important to recognise because this often limits the amount of bulk reduction that can be achieved, and a patient’s expectations should be managed accordingly.

Indications for treatment include mass effect, recurrent infections or bruising, or functional issues. Macrocystic disease is relatively easily treated with percutaneous sclerotherapy; doxycycline is usually the first line agent, as it is widely available and has a high safety profile, though STS is probably just as effective. There is good date available on doxycycline sclerotherapy outcomes (Alomari and Dubois 2011; Shergill et al. 2012). OK-432 (Picibanil, Chugai Pharmaceuticals, Tokyo, Japan), a lyophilised form of group A *streptococcus pyogenes* incubated with benzylpenicillin, was widely used as a sclerosant for LMs but far less commonly available now.

**Fig. 10:**
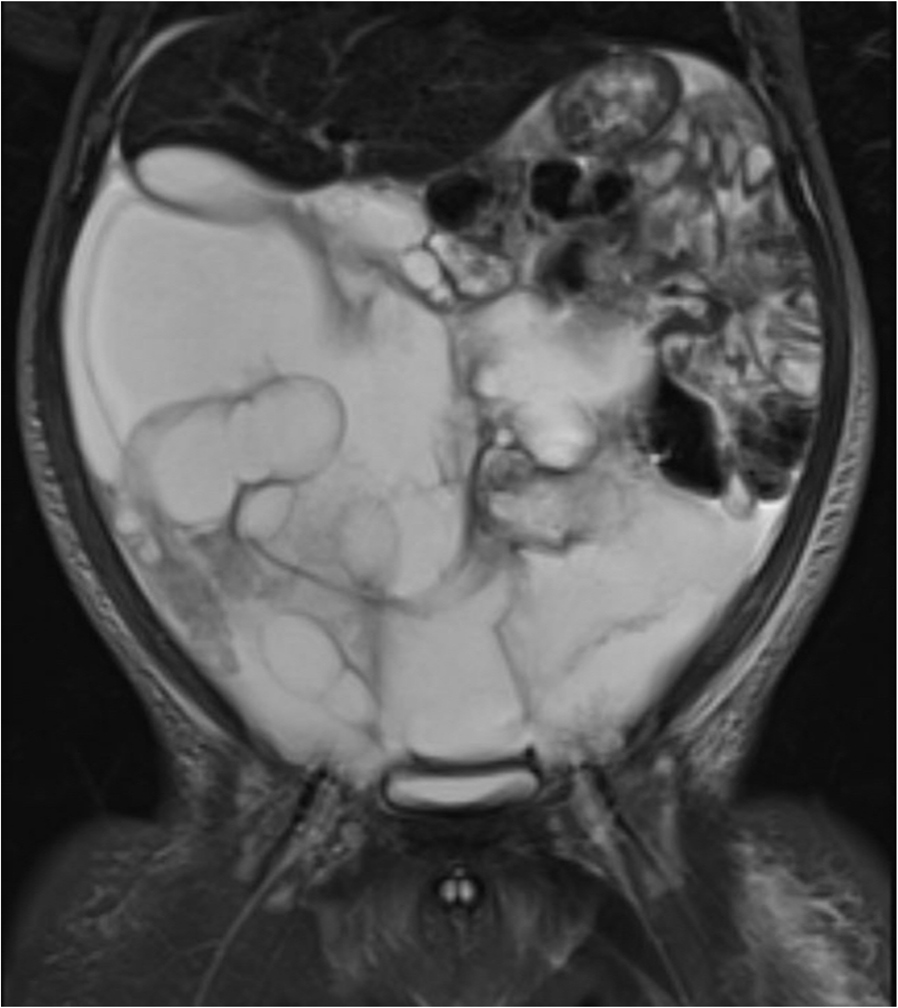
Coronal T2 weighted MR image of a large mesenteric lymphatic malformation in a 2 year old male. This lesion would be suitable for serial sclerotherapy via 2–3 pigtail drains

Sclerotherapy of LMs is usually straightforward. Macrocysts should be drained to almost-complete dryness. The dose of doxycycline to instil depends on patient size more than the volume of fluid drained. Most operators recommend doses of around 100-200 mg doxycycline per procedure in infants, 300-500 mg in young children and up to 1200 mg in teenagers (Hawkins and Chewning 2019). Haemolytic anaemia, metabolic acidosis and transient hypoglycaemia has been described in babies after systemic doxycycline absorption (Coughlin et al. 2019). Blood glucose levels should be monitored in children under the age of 1 year until they start to feed again (Cahill et al. 2011). A dual-agent technique can be used in lesions that don’t respond well to simple doxycycline sclerotherapy. This involves instilling STS or ethanol into the cysts and leaving it to dwell for 5–10 min before aspirating it and instilling a standard dose of doxycycline, the theory being that the STS denudes the endothelial lining of the cysts and allows the second agent to be more efficacious (Shiels et al. 2009). In general, sclerosing agents do not need to be foamed, as 360^0^ wall contact is more easily achieved in collapsed macrocysts than in VMs. Simple macrocystic lesions may only require one treatment; complex lesions may require a series of procedures. Intensive treatment of very large macrocysts, such as those seen in mesenteric LMs or neonatal cervicofacial malformations, can be achieved if pigtail drains are inserted at the time of the first procedure; serial sclerotherapy procedures can then be performed on the ward over several days with the child awake (Fig. 10).

Microcystic disease is notoriously difficult to treat. Bleomycin is generally believed to be the best agent to achieve bulk reduction in relatively solid lesions (Chaudry et al. 2014). The aim is to bathe the entire area with bleomycin, even aiming to instil the drug into the stroma or solid component of the lesion where it may be more effective. This can be done under US guidance. Some operators mix bleomycin with 20% albumin solution or other agents with the aim of thickening it to prolong its dwell time. Most oncologists and pharmacists would prefer to avoid the use of cytotoxic drugs in children under the age of one and dose limits should be respected in older children (a maximum of 15,000 IU per procedure at any age and a maximum life time dose of 2–3000 IU per kg up to a maximum of 80–100,000 IU). Whether to monitor respiratory function or not in these patients is debateable but families should be made aware of the risk of pulmonary fibrosis with the use of this drug (Zorzi et al. 2015). Although it is unlikely that much, if any, of the active drug will be absorbed systemically when used in lymphatic disease, it is good practice to avoid supplemental oxygen unless clinically needed, as it is thought to increase the risk of pulmonary fibrosis. Flagellate hyperpigmentation is well-documented with the systemic use of bleomycin and efforts should be made to avoid adhesives during the procedure, including ECG monitoring pads. Patients should be advised not to scratch the skin for 48 h. Anything stuck to the skin should be left in situ for 48 h, until bleomycin is washed out of the dermis, or removed with appropriate solvents. Finally, sporadic cases of acute bleomycin toxicity and death have been reported (Cho et al. 2020).

Small children with LMs will of course require general anaesthesia (GA) for sclerotherapy but non-GA sclerotherapy is an excellent option for older children. Note that both doxycycline and bleomycin sting on injection. To avoid this, macrocysts can be pre-treated with a small dose of local anaesthetic prior to injection of doxycycline. Mixing bleomycin with a small dose local anaesthetic prior to injection reduces the stinging sensation.

As always with malformations, the challenge is knowing when to intervene. There are a small proportion of LMs that shrink spontaneously in the first few months of life, which should prompt IRs to consider delaying treatment of asymptomatic lesions in young babies. It has been suggested that general anaesthesia in the first year of life may have long-term consequences on a child’s development, which is another good reason to delay treatment of asymptomatic lesions (McCann and Soriano 2019).

There is often a high level of concern regarding the potential for post-operative swelling after sclerotherapy of head and neck lesions necessitating a period of elective intubation after each procedure. In the authors’ experience, this is rarely the case as long as each case is treated with appropriate caution and by an experienced team with on-site ENT cover.

Lymphatic malformations of the tongue are a management challenge. Involvement may be purely mucosal, resulting in a tongue surface which is prone to bleeding, infections and irritation with spicy foods, or the malformation may involve the deeper tissues of the tongue, resulting in macroglossia. ENT involvement is paramount. Topical coblation of superficial lesions is highly effective in re-surfacing the tongue with healthier tissue. Tongue reduction surgery is a viable option in an experienced surgeon’s hands, but many operators report excellent results with bleomycin sclerotherapy (Parashar et al. 2020). The technique involves traction on the tongue followed by ultrasound guided infiltration of the microcystic disease using 3000–5000 IU bleomycin per treatment over a series of repeat procedures.

**Fig. 11:**
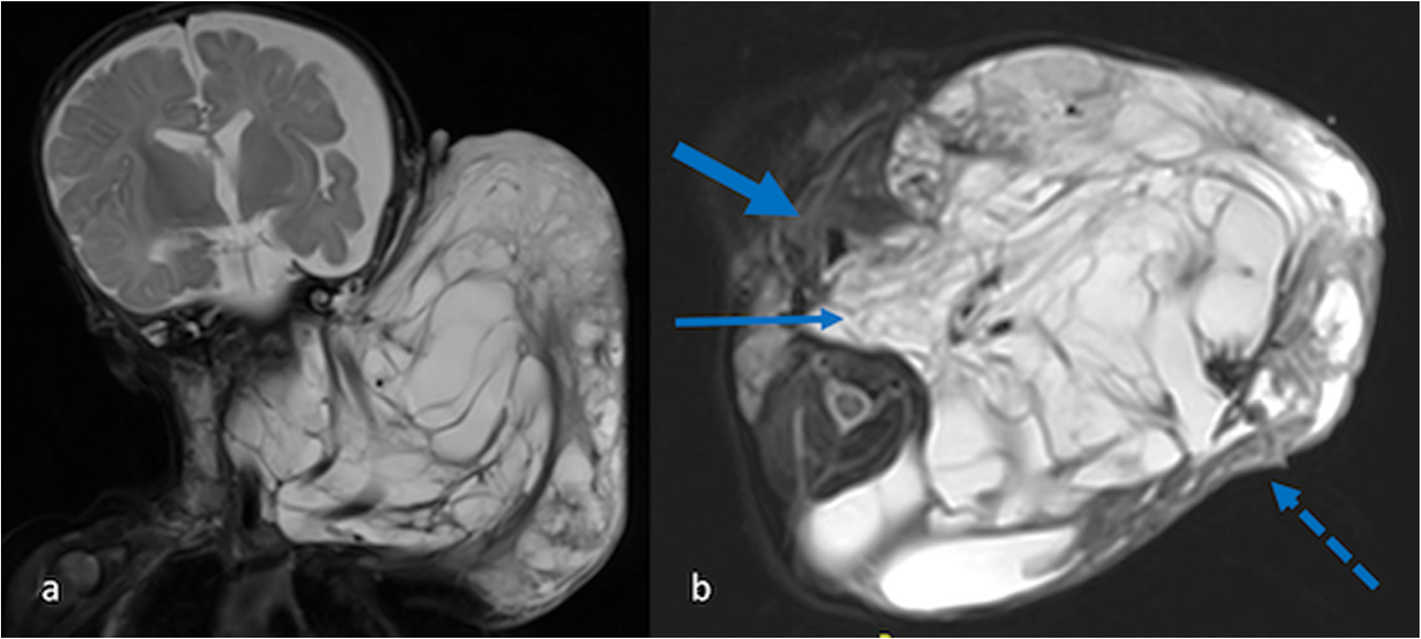
Lymphatic malformation in a 2 day old male. **a** coronal and **b** axial T2 weighted MR images delineate the complex lesion causing marked distortion of the anatomy of the neck. On the axial image, the prevertebral disease (thin arrow) displaces the trachea (thick arrow); this is the area that should be targeted with sclerotherapy to improve the child’s respiratory status. The more bulky, superficial disease (broken arrow), although tempting to treat, is of little clinical consequence at this stage

Some of the most challenging cases are the complex, extensive cervicofacial LMs that present at birth with airway compromise. Most are diagnosed antenatally and should be considered for an EXIT delivery (Shamshirsaz et al. 2019). The IR team should be involved from the start. The approach depends on the local multidisciplinary team but serial sclerotherapy is an excellent option to get a child off the ventilator and partially debulk the lesion. The aim should be to shrink the lesion enough to allow the child to move their head and to feed, so that they can put on weight and thrive prior to elective surgery a few months later. Although it is tempting to target superficial macrocystic disease, it is often the deep component that is causing functional issues (Fig. 11).

LMs are a result of somatic PIK3CA mutations and therefore sirolimus is effective in managing some types of lymphatic disease (Castillo, Wiegand). There is still little data on which lesion types respond best to mTOR inhibitors or on appropriate dosing regimens. Disease often recurs once the drug is stopped and side effects are not insignificant (Weigand). Because of these factors, sirolimus use is usually restricted to extensive or complex disease, including generalised lymphatic anomaly (GLA) (Ricci et al. 2019).

Finally, surgical debulking of complex lymphatic disease can often lead to the formation of post-operative seromas. This shouldn’t necessarily be seen as a complication of treatment and can often be predicted. Surgeons should be made aware that these seromas can be well managed via sclerotherapy through the surgical drains, a procedure that can be performed as required, on the ward or as an outpatient.

### Arteriovenous malformations

Although arteriovenous malformations (AVMs) are present from birth, it is relatively rare for them to cause clinical problems during childhood. As with adult cases, IRs often face a management dilemma once an AVM is diagnosed in a child, because the risks of treatment are usually significant. There are often good reasons to delay treatment until the lesion becomes symptomatic, but this may lead, inadvertently, to a situation where intervention is delayed until the lesion becomes much more difficult to treat. Some, but not all, childhood AVMs can experience periods of rapid growth, particularly around puberty, although it is difficult to predict which lesions will behave this way complicating the decision of when to best intervene. In children a common, significant long-term effect of an AVM which is centred near around a growth plate relates to its interference with bone growth. These cases may merit early intervention even if the child is asymptomatic. Embolisation of these high flow lesions can also lead to arrested or asymmetrical growth, which may then require subsequent orthopaedic correction.

The approach to embolisation of high flow lesions outside of the neuro-axis in children is very similar to that in adults and beyond the scope of this paper. Conservative dose limits should be maintained when using ethanol as an embolic agent in children. It is highly effective in treating high flow lesions, which is thought to be due to its efficacy in destroying, rather than merely damaging, endothelial tissue and hence eradicating the production of angiogenic growth factors (Hawkins and Chewning 2019; Yakes 2004). Keeping to a cautious dose limit of 0.5 mL/kg per session is recommended (Hawkins and Chewning 2019). Caution, too, should be used with ethylene vinyl alcohol suspended in dimethyl-sulfoxide (Onyx™, Medtronic, Dublin, Republic of Ireland). On injection, the dimethyl-sulfoxide (DMSO) washes out of the solution and into the bloodstream, allowing Onyx™ to polymerise. Systemic DMSO is excreted via the lungs after administration. Cases of DMSO toxicity, including life threatening pulmonary oedema, haemolysis and renal failure, have been reported after Onyx injection and may be dose related (Ashour et al. 2012).

Recently, interstitial bleomycin injection has been suggested as a means of down-regulating symptomatic AVMs (Jin et al. 2018), though little is yet understood about the mechanism of action of bleomycin in this setting.

#### Fibroadipose vascular anomaly FAVA

**Fig. 12:**
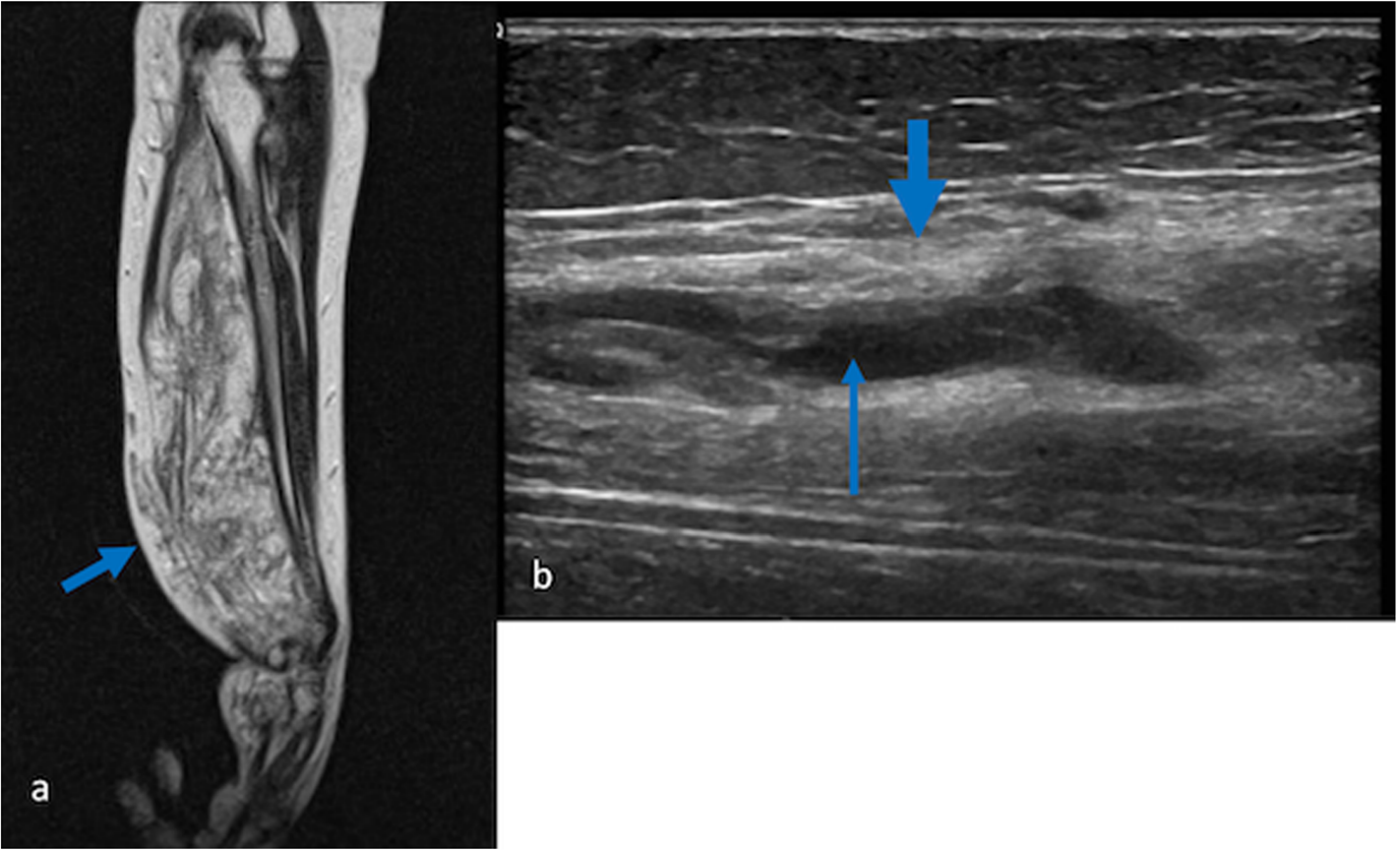
**a** sagittal T1 weighted MR image of the forearm in a 12 year old female. The FAVA lesion (short arrow) replaces almost all of the flexor compartment and is causing contracture at the wrist; **b** US image of a FAVA lesion of the calf, showing marked echogenicity of the affected muscle (thick arrow) and central dysplastic veins (thin arrow)

Fibro-adipose vascular anomaly (FAVA) is an unclassified type of vascular anomaly that results from somatic mutations of the PIK3CA pathway (Luks et al. 2015). It is a relatively newly described condition, first described in 2014 as an intramuscular lesion in which fibrofatty infiltration of muscle is associated with dysplastic venous channels (Fernandez-Pineda et al. 2014; Alomari et al. 2014). Previously these lesions were most commonly diagnosed as venous malformations with fatty overgrowth or “intramuscular haemangiomas” (Alomari et al. 2014). FAVA typically presents as swelling of part of a limb, often the calf, in school-age children. It is associated with significant pain, beyond that expected with a common VM, and over time, it often causes muscle contractures that are highly resistant to therapy (Alomari et al. 2014). The diagnosis can be confirmed by ultrasound and/or MRI, both of which can identify the characteristic pathological elements of the lesion (Alomari et al. 2014; Cheung et al. 2020) (Fig. 12). Pathologists can hesitate to make a diagnosis of FAVA on biopsy, based on its rather non-specific histopathological features, but biopsy can be helpful in excluding other diagnoses such as sarcoma.

Medical management of FAVA is in its infancy. Because the effects of *PIK3CA* mutations are at least partly mediated by mTOR, sirolimus therapy has been tried with some success (Erickson et al. 2017). It is generally agreed that sclerotherapy is ineffective in managing the pain associated with FAVA; closure of the dysplastic veins does little to alleviate the pain, swelling or contractures. A proportion of lesions are progressive, so early intervention should at least be considered before too much muscle bulk or more than one compartment is involved. Cryoablation appears to be an effective treatment for pain and may improve functional symptoms (Shaikh et al. 2016); in extensive lesions, it should be targeted at the regions of maximal pain. Localised lesions may be better suited to surgical resection, though this comes with an inevitable trade-off in terms of function if an entire muscle compartment requires debulking (Cheung et al. 2020, Wang et al. 2020). Recurrence rates are not yet understood.

For each of these pathologies, it is clear that there is no one treatment pathway that fits all, a fact that is as frustrating for clinical teams as it is for families. To compound this, there is very little, if any, hard evidence for the efficacy of the treatments discussed here. For this reason alone, it is essential that teams use standardised outcome measures to gather some evidence of efficacy for individual patients, for specific interventions and for different disease entities. The OVAMA project is working towards uniform outcome reporting in clinical research on peripheral vascular malformations (Lokhorst et al. 2019). Tools such as quality of life (QoL) assessments also help children and families to give structured feedback and gain some personal objectivity in the treatment of their condition.

### Mixed lesions and overgrowth syndromes

Vascular malformations can occur as part of a much wider abnormal growth pattern. A huge array of malformations can co-exist in this way but there are some patterns that are seen more commonly than others. Historically, these have been given a variety of names, many of which are eponymous, very poorly defined and widely misused, such as Klippel-Trénaunay syndrome and Proteus syndrome. Such nomenclature has led to years of confusion in the medical community regarding the diagnosis and, much more importantly, treatment of these conditions. It is now better understood that almost all of these ‘syndromes’ or clusters of malformations are related to underlying somatic mutations in growth genes (Uller et al. 2014). The most common mutation currently recognised lies in the phosphatidylinositol-4,5-bisphosphate 3-kinase, catalytic subunit alpha (PIK3CA) gene, giving rise to abnormal PIK3-AKT-mTOR pathway activations, leading to dysregulation of cell signalling and angiogenesis (Kang et al. 2020). Mosaic somatic mutations in PIK3CA underlie many of the complex overgrowth syndromes; the use of the umbrella term ‘PIK3CA-related overgrowth syndrome (PROS) is very helpful in simplifying the diagnostic and management approach to these seemingly heterogeneous disorders and should be promoted. Recognition of the underlying PIK3CA mutations is of significance on two counts; it suggests that medical therapies can be used to target overgrowth at a cellular level, and it hopefully encourages a more simplistic and methodical approach to treatment, recognising that the condition as a whole cannot be cured by any one surgical or IR intervention.

Klippel-Trenaunay Syndrome (KTS) is the most well-known of the overgrowth syndromes, characterised by unilateral limb overgrowth, a capillary malformation of the skin (‘port wine stain’) and slow-flow vascular malformations (Uller et al. 2014). A dysplastic embryonic marginal venous system is often present. It should be noted, however, that this label, like that of Proteus, is vastly over-used in patients with other forms of overgrowth and is often unhelpful. Limb overgrowth can have a myriad of underlying components. Patients with complex overgrowth require a simple label, such as PIK3CA-related overgrowth spectrum or PROS, and a truly multi-disciplinary approach. Management should be carefully tailored according to the patient’s individual needs. This may include endovascular closure of embryonic veins, sclerotherapy of low flow malformations, compressions garments, CO2 laser of skin lesions, surgical management of gastrointestinal or pelvic disease, and orthopaedic involvement for the management of overgrowth.

Sirolimus has a role in the treatment of both PIK3CA-related overgrowth and non-PROS growth disorders but it is clear that such medical therapies are still in their infancy (Adams et al. 2016; Horbach et al. 2016). More specific gene mutations need to be identified so that the vast range of differing phenotypes can be targeted more effectively.

Vascular malformations occurring as part of a wider syndrome can be challenging to treat, occurring in difficult-to-reach sites and often presenting late. These can include the urogenital tract, GI tract, mesentery, bone marrow and orbit. As with so many other conditions, it often falls to IR to suggest minimally invasive and innovative interventions and encourage collaborative approaches to treatment. These include endovenous laser therapy (EVLT) for malformations with a predominantly ‘dysplastic-vein’ type anatomy and cryoablation for more solid overgrowth lesions (Cornelis et al. 2017; Shaikh et al. 2016; King et al. 2013). Endoscopically-guided sclerotherapy of the bladder, mesentery and airway is both effective and extremely attractive to patients, compared to aggressive surgical options. We owe it to these patients to think collaboratively and push the boundaries of what is possible (Barnacle et al. 2016; Sinha et al. 2013; Stimpson et al. 2012).

## Conclusion

There are some key differences in the approach to vascular anomalies in children. IRs play a crucial role in ensuring the commonest, self-limiting vascular tumours are left alone, while the rare aggressive life-threating types receive emergent embolisation and diagnostic biopsy when required. The IR approach to treating vascular malformations in children must include a working knowledge of the sclerosant agent doses permitted in small patients and the wider effects of severe disease, including coagulopathy and joint destruction. Overgrowth, which is often seen as part of this spectrum of conditions, is now better understood than ever. It is the role of the IR to apply a simple and practical approach to the components of these conditions that we can treat and not overcomplicate matters. Most IRs recognise the challenge of treating patients with vascular anomalies. Where this involves children and families, an integrated approach that involves a strong multidisciplinary team and a long-term view, is just as important and perhaps even more rewarding.

## Data Availability

Not applicable.
